# A qualitative evaluation of stakeholder perspectives on the implementation of HIV services within adolescent and youth-friendly services for the youth in Nampula, Mozambique

**DOI:** 10.1186/s12913-025-13414-0

**Published:** 2025-09-30

**Authors:** Phepo Mogoba, Joana Falcao, Tsidiso Tolla, Allison Zerbe, Eduarda Pimentel De Gusmao, Landon Myer, Elaine J. Abrams

**Affiliations:** 1 Division of Epidemiology & Biostatistics, School of Public Health, University of Cape Town, Level 5, Falmouth Building, Anzio Road, Cape Town, South Africa; 2 Mailman School of Public Health, ICAP at Columbia University, New York, NY USA; 3 Division of Social and Behavioral Sciences, School of Public Health, University of Cape Town, Cape Town, South Africa; 4 Department of Pediatrics, Vagelos College of Physicians and Surgeons, Columbia University, New York, NY USA

**Keywords:** Adolescents, Young adults, HIV services, Youth-Friendly services, Implementation, Mozambique

## Abstract

**Background:**

Despite the well-documented challenges affecting HIV treatment for adolescents and young adults living with HIV (AYAHIV) in low- and middle-income countries, the implementation of recommended Adolescent and Youth-Friendly Services (AYFS) remains limited. In this qualitative study, we explored stakeholder perspectives on the implementation of HIV services for AYAHIV within AYFS in Nampula, Mozambique. This study forms part of a broader investigation on improving HIV care among adolescents and young adults living with HIV (AYAHIV) in Mozambique.

**Methods:**

Using a purposive sampling strategy in an exploratory qualitative research study, we conducted semi-structured in-depth interviews between September and December 2021 with stakeholders, including healthcare workers (HCWs) delivering HIV services and key informants managing HIV services for youth on ART in 12 health facilities participating in the CombinADO study. The interviews were analyzed using thematic analysis.

**Results:**

Participants included 59 stakeholders (44% HCWs and 56% KIs), the majority of whom were female (71%). The findings suggested variations in stakeholder perspectives on HIV service delivery, indicating disparities in service quality across different health facilities. While positive assessments prevail, notable barriers include inadequate staff training, staff shortages, limited privacy, absence of caregiver support, insufficient HIV literacy among AYAHIV and caregivers, and access constraints. Facilitators include patient-provider relationships, collaborative care, well-trained HCWs, support groups, effective dissemination of HIV awareness information, one-stop shops, and privacy.

**Conclusions:**

Stakeholders shared positive perceptions and experiences concerning the delivery of HIV services to AYAHIVs within the current AYFS framework in Nampula. However, they also identified barriers—such as limited staff capacity, insufficient training, and lack of private spaces—that hinder the effectiveness of these services in addressing challenges related to retention, ART adherence, and HIV stigma among AYAHIVs. Addressing these barriers is essential to optimize HIV service delivery and improving outcomes for adolescents and young adults. Notably, findings from this study informed the refinement of the CombinADO intervention, which integrated enhanced HCW training, infrastructure strengthening, and peer-led support to better respond to AYAHIV needs within AYFS.

**Trial registration number:**

ClinicalTrials.govNCT04930367. Registered on 18th June 2021.

**Supplementary Information:**

The online version contains supplementary material available at 10.1186/s12913-025-13414-0.

## Background

Adolescents and young adults living with HIV (AYAHIV) in low- and middle-income countries often encounter several well-documented multi-level challenges that significantly impact their adherence to ART and their ability to remain engaged in HIV care programs [1–5]. Due to the specific care needs linked to the developmental stage of adolescence and early adulthood, this population requires tailored strategies that would support their HIV care treatment and address these challenges effectively [6].

Adolescent and youth-friendly services (AYFS) are recommended by the World Health Organization (WHO) for addressing key health priorities, including those related to HIV, among adolescents and young adults (aged 10–24 years). The AYFS model incorporates principles of equity, accessibility, acceptability, appropriateness, and effectiveness, designed as a healthcare model to provide a safe, friendly, and supportive environment for AYAHIV [7]. It offers a one-stop shop model to provide adolescent-friendly sexual and reproductive health services integrated into HIV treatment and care. The recommended best practices for AYFS emphasize engaging young people in program design and implementation, enhancing service accessibility, ensuring affordability, providing alternative ways to access services, training staff on AYFS, and incorporating peers in service delivery [7]. Consequently, integrating HIV services into AYFS offers an opportunity to address barriers faced by AYAHIV in accessing quality health services to support retention and adherence to ART [8]. While there is favorable evidence regarding the impact of AYFS on HIV outcomes [8–12], it is worth noting that there is also evidence of multilevel challenges regarding the implementation of AYFS [13–19]. A recent qualitative study conducted among healthcare providers in Ethiopia illuminated numerous barriers to AYFS access at various levels—provider, health facility, adolescents’ community, and the broader health system [20]. Similarly, a qualitative study evaluating provider perspectives on AYFS provision in South Africa revealed that providers believed these services were under-implemented, attributing this to barriers such as insufficient youth-friendly training among staff and lack of designated spaces for young people [14]. These challenges might make it more difficult to achieve expected health outcomes using AYFS. Therefore, it becomes imperative to gather contextual evidence at the country level to identify and develop effective strategies to overcome implementation challenges. Such efforts are necessary to overcome any identified implementation challenges to ensure quality services can be available to meet the specific needs of the adolescent and young adult population.

In Mozambique, it is estimated that there are 140,000 adolescents (aged 10–19 years) and 320,000 (aged 15─24) living with HIV [21, 22]. In 2020, 40% of all new infections (39,000) occurred in adolescents, and 72% of these were in girls and young women [23, 24]. There are substantial gaps along the HIV care continuum for adolescents as well, with viral suppressions low among adolescents and young adults, at 45% among adolescent girls and young women and 42% among adolescent boys and young men (compared to 64% among all people living with HIV) [25]. National adolescent HIV guidelines endorse youth-friendly approaches as critical to improving service uptake and retention; however, implementation of AYFS remains limited [26, 27] due to resource constraints, inadequate infrastructure, and lack of prioritization of care for AYAHIV.

Expanding upon earlier formative work conducted in the CombinADO study (NCT04930367) to identify service needs among AYAHIV, ICAP collaborated with the Ministry of Health (MISAU) to develop comprehensive, multi-component, multi-level strategies aimed to improve viral suppression, retention, and ART adherence among AYAHIV by enhancing HIV care services within AYFS in Nampula province, in northern Mozambique [28–31]. Given the study’s objectives, this work was done to understand experiences and perceptions regarding HIV service delivery to AYAHIV within AYFS to inform the implementation of CombinADO study.

Hence, this paper presents perspectives of stakeholders working within services at health facilities participating in the CombinADO study. This study’s objectives include: (1) Describing stakeholder perspectives on the pre-study implementation status of HIV services for AYAHIV within AYFS and (2) Assessing stakeholders’ perceptions of barriers and facilitators influencing HIV care service implementation, utilization, and effectiveness when provided within the AYFS. By examining stakeholder insights across a diverse range of health facilities, this study contributes to the growing body of literature on contextual implementation barriers and facilitators for adolescent HIV services. It provides operational evidence to guide future scale-up of youth-centered HIV interventions in Mozambique and similar settings.

## Methods

### Study design

This work was done in the preparatory stages of the CombinADO trial [29]. The study used an exploratory qualitative research design, utilizing semi-structured in-depth interviews (IDIs) to collect data from stakeholders that included healthcare workers (HCWs) and key informants (KIs). A thematic analysis approach was used to analyze and present the data [32], describing participants’ experiences and perceptions regarding the HIV services within AYFS, prior to the implementation of the CombinADO study.

### Study setting

This study was conducted in health facilities in Nampula province, a low-resourced region in Northern Mozambique. The HIV prevalence among young people aged 15–24 years in Nampula is estimated to be 4.1%; yet only 16% of clinics offer AYFS [27]. HIV services offered in AYFS represent a sector within each health facility offering sexual and reproductive health services exclusively for adolescents and young people aged between 10 and 24 years. The 12 participating health facilities were randomly selected from 15 eligible sites by an independent biostatistician for inclusion in the CombinADO study. Selection criteria included proximity to Nampula City (within a 4-hour drive), a minimum of 220 AYAHIV aged 10–24 years currently on ART, and implementation of a one-stop-shop AYFS model of care. Approximately 10,370 AYAHIV receive routine ART at various HIV service sites across the 12 participating health facilities. Of the selected facilities, six were urban, four were rural, and two were semi-urban.

### Sampling

A purposive sampling strategy was used to ensure representation HCWs delivering HIV services for youth on ART in CombinADO included facilities and KIs managing HIV services at regional and national levels. For each of the 12 health facilities participating in the CombinADO study we invited the health facility director and asked him/her to provide the names of all HCWs involved in HIV services provision for AYAHIV at the facility. Furthermore, invitations were extended to the district health directors where the CombinADO study would be implemented, the provincial health director, and a MISAU focal point for the HIV program. All the participants were explained that their participation was completely voluntary with no repercussion on their professional roles and that all the results will be anonymized. All invited stakeholders agreed to participate in the study. The sum of the stakeholders that accepted to participate was considered our total sample size as it captured a diversity of stakeholders’ roles and experiences associated with the CombinADO study implementation.

## Study instrument

Interviews were conducted using a semi-structured IDI guides developed by the study team for this study (See Supplementary file 1). The guides were carefully reviewed and refined through extensively discussed within the study team to ensure alignment with the study objectives and contextual appropriateness. While only minor adaptations were made to the guides to accommodate differences in stakeholder levels (e.g., HCW vs. KI participants), the core questions included in this analysis were consistent across all participants. The questions explored concepts regarding the implementation of HIV services for AYAHIV within AYFS before the CombinADO study, including those that addressed: (1) participants’ rating of HIV services for AYAHIV at their respective health facilities on a scale of 1–10, with 1 meaning that services were working poor and 10 meaning that the services were excellent; (2) facilitators and barriers to the implementation of HIV services for AYAHIV; and (3) perceived impact of HIV services on retention, ART adherence, and HIV stigma for AYAHIVs which were among the aspects targeted by the trial’s intervention materials.

### Data collection

The interviews were conducted in Portuguese between September to December 2021. All interviews were completed in-person by a Mozambican interviewer, with no prior relationship with participants, and extensive experience in social research techniques and training in research ethics. Interviews took place in private rooms within health facilities or on-site offices. Each interview lasted 45–60 min. All participants gave written informed consent prior to participation. All the interviews were audio-recorded with participants’ permission.

### Data management and analysis

All recorded sessions were transcribed verbatim in Portuguese by an independent transcriber. The transcripts were checked against the audio recordings to ensure their accuracy and anonymization and then were translated from Portuguese into English by a private organization outside the study team. Thematic analysis was used to identify patterns in the interview transcripts. Thematic analysis was conducted by JF and TT using the Braun and Clarke guidelines [32]: familiarizing oneself with the data, generating initial codes, searching for themes, reviewing themes, defining and naming themes, and producing the results report. An initial set of transcripts were analyzed to generate initial codes. This analysis employed a dual approach, combining deductive coding based on the interview questions and inductive coding based on the participants’ responses. After highlighting keywords and phrases while reading the transcripts, these notes were then compared among JF and TT, transcribed onto coding sheets, and grouped into overarching themes. Themes were compared to minimize interpretive bias. Discrepancies between JF and TT were discussed and adjusted until a consensus was reached. Final themes were refined and defined through extensive discussion among JF and TT and the first author (PM).

Although participants were sampled from both facility-based and stakeholder-level roles, subgroup sizes were insufficient to allow for systematic comparison. As such, themes were derived from a combined analysis of all participant perspectives. Even though the Social Ecological Model (SEM) was not used to guide the study design or interview development, it was applied as an interpretive lens during the results synthesis. The SEM helped situate identified barriers and facilitators at multiple levels — individual, interpersonal, organizational, and community — offering a layered perspective on the multilevel factors shaping HIV service delivery for AYAHIV within AYFS. This framing helped enhance the clarity and organization of the themes. JF is a Mozambican psychologist, epidemiologist and researcher with expertise in project implementation and research in a range of public health issues in Mozambique. TT is a social and behavioral sciences researcher whose work focuses on masculinity and HIV, adolescent sexual and reproductive health, intimate partner violence, and community engagement. PM is an epidemiologist with her research focus on adolescent, maternal and child health, and HIV, and was pursuing a PhD in implementation science at the time of this study. Translated transcripts were uploaded and analyzed using NVivo 12 software. All data were stored securely on password-protected systems at Columbia University and only accessible to authorized members of the study team through approved data request procedures.

### Ethics considerations

The study was approved by the Mozambique Ethics and Scientific Review Committee (CNBS) and the Columbia University Irving Medical Center Institutional Review Board. The quotes are attributed to participants using anonymized identifiers to protect the confidentiality of the respondents. The coding system is structured as follows: stakeholder type (e.g., KI or HCW)/facility code (e.g. AA, LL)/participant type sequence number within that facility (e.g., 001, 002).

## Results

A total of fifty-nine participants were interviewed (Table 1). The majority were health facility staff engaged in providing HIV services within AYFS clinics for AYAHIV. Three stakeholders represented provincial health services, and one was from the MISAU. Results are organized according to the following four themes: (i) current state of HIV services (before CombinADO trial implementation); (ii) facilitators for the delivery of HIV services; (iii) barriers impacting the delivery of HIV services to AYAHIV within AYFS; and (iv) participants’ perceived efficacy of HIV services to impact on retention, ART adherence, and stigma.

**Table 1:** Description of study participants

Description of study participants, *N* = 59		
	*n*	%
Type of Participants		
Health Care Workers	26	44%
Key Informants	33	56%
Gender		
Female	42	71%
Male	17	29%
Level		
Health Facility level	55	93%
Provincial level	3	5%
Minister of Health level	1	2%

### Current state of HIV services for AYAHIV within AYFS

Generally, the perceptions of HCWs and KIs on the implementation of HIV services for AYAHIV painted a picture that the services were working efficiently despite existing challenges. Largely, participants gave scores ranging between seven and ten.

*I would rate it 10 because there is already a service in place to follow up adolescents and we already have trained professionals to follow up each adolescent living with HIV. (HCW.II.001)*

*I would give an 8. Regarding the [HIV] consultations I think it is happening well and now*,* what we are lacking is to achieve adolescents’ [viral] suppression*,* some of them still can’t accept what their serostatus is*,* some of them don’t want to take the medicine*,* some of them come to the health facility and some of them don’t. So*,* I think it is still a challenge but based on those resources that we have in the health facility*,* we are trying our best to try to get the adolescents to be able to come into the health facility and get the package that we deliver here*,* that is what we are doing. (KI.UU.001)*

Nevertheless, some participants gave ratings of five or less, reflecting concerns about systemic limitations, fragmented support across sectors, and the perception that adolescent HIV services were still underdeveloped or inadequately integrated.

*I think I would give it [a score of] 5… effectively the adolescent issue is not something that only stays within the health facility … I don’t think we are there yet. (KI.GE.004)*

Interestingly, some inconsistencies were observed between the numeric ratings and the accompanying narrative justifications. A few participants assigned high scores while simultaneously describing persistent challenges related to infrastructure, human resources, and adolescent engagement.

*Well*,* what is not working is that we have poor adherence… although we have a private place of the* AYFSs, *the conditions are not the best… Then we have the human resources… and the work is intense.* (KI.OO.001)

*I would give a score of 10… the staff involved in implementation is already trained [but we still have] deficiencies.* (KI.LL.001)

These inconsistencies highlight the complexity of assessing perceived service quality in implementation research. While stakeholders generally acknowledged progress, they also revealed areas where improvements are still urgently needed, especially regarding workforce, infrastructure, and adolescent engagement.

### Facilitators for the delivery of HIV services to AYAHIV within AYFS

Participants identified a range of factors that facilitated effective provision of HIV services for AYAHIV. The facilitators operated at multiple levels and often worked in combination to promote engagement with services and adherence. Table 2 displays a summary of the major facilitating factors that participants identified as well as illustrative quotes alongside each facilitator. At the individual level and interpersonal levels, the ability of HCWs to respond to the needs of adolescents were seen as essential to building trust and continuity of care. Availability of HCWs and peer educators trained in adolescent-responsive care were also seen as important enablers, particularly when they were perceived as approachable and consistent. Furthermore, involvement of caregivers in the care of adolescents and the existence of support groups contributed to adherence by reinforcing social support and treatment literacy. At the community level and system levels, stakeholders highlighted the value of collaborative efforts with schools and community-based organizations, along with campaigns via media and outreach. Such efforts supported normalization of HIV care and increased demand. Additionally, structural factors such as private consultation spaces and the integration of services through one-stop-shop models were cited as critical to improving service accessibility and adolescent comfort.

**Table 2:** Participant views on facilitators to delivery of HIV services to AYAHIV within AYFS

Theme	Illustrative quote
Building strong personal relations with AYAHIV	*“… even though we have so many (AYAHIV) enrolled*,* but I know all of them… we say: the services that you need are here and even if it is not your appointment day*,* if you have a concern*,* don’t be limited*,* come to us and we will take care of you”* (HCW.NN.002)
Collaboration with schools and community partners	*“We have some partners… we have also been doing mobile brigades in the schools*,* and this is also facilitating a lot”* (HCW.AA.002)
Availability of HCWs and peers trained on adolescent health and with the capacity to work with adolescents and their complexity	*“I think it is basically those HCWs that are skilled in dealing with adolescents*,* and the peer educators…”* (KI.AA.010) *“We have peers that have been doing home visits… they are the ones who go out there and motivate those teenagers”* (HCW.LL.002)
Creation of support groups for AYAHIV	*“Doing little groups with those AYAHIV… and make experiences exchange.”* (HCW.SS.003) *“I think that these peers and support groups have helped us a lot in the retention of teenagers living with HIV”* (HCW.LL.002)
Caregivers’ engagement in AYAHIV ART	*“Improved communication and interaction between caregivers and adolescents…”* (KI.LL.001)
Using peers to support HIV treatment among AYAHIV	*“We have community peers and health facility peers*,* so for those adolescents who are late in getting their (HIV) medication or in coming for their consultation we have immediate intervention (phone calls and home visits)*,* that is why we are managing to control the defaulting problem”* (HCW.SS.001)
Dissemination of HIV information and HIV services withing the health facilities, in the communities and through the media	*“The awareness… on the radios*,* on the televisions*,* in the communities… the lectures that we give… this is what is facilitating the adherence*,* even if it is not much*,* but it facilitates the adherence of the few who come here”* (KI.EE.002)
One-stop model and private consultation rooms to improve access to HIV services	*“… the one-stop concept is working… I think that is a big gain” (KI.GE.003)* *“What is making it easier? First thing is having a proper consultation room with privacy”* (HCW.EE.003)

### Barriers impacting the delivery of HIV services to AYAHIV within AYFS

Despite generally positive perceptions, participants identified several barriers that hindered the provision of HIV services to AYAHIV within AYFS. Table 3 details the main barriers identified by participants together with illustrative quotes. These included system-level, interpersonal, and structural challenges that undermined consistent, high-quality service delivery. Health system constraints such as shortages of staff and their inadequate training were reported to affect both the scope and quality of services, particularly the delivery of psychosocial support. Poor infrastructure and a lack of private space for consultations further compromised confidentiality and comfort for adolescents. Low levels of HIV literacy among AYAHIV and caregivers were flagged as contributing to poor adherence and disengagement from care, especially in cases where adolescents were unaware of their HIV status due to delayed disclosure.

**Table 3:** Participant views on barriers to delivery of HIV services to AYAHIV within AYFS

Theme	Illustrative quote
Human resources –shortages and untrained HCWs	*“… there are times that we don’t have enough staff… the follow up of certain indicators for HIV also ends up being deficient… if we had more [staff]*,* it would be better*,* the follow up would also be better” (KI.SS.001)* *“… the training of the HCWs is not fine… Sometimes the clinical consultation is done looking at the context of antiretroviral treatment and the other psychological aspects go unnoticed*,* because these HCWs need training… to be able to take care of these situations” (KI.SS.002.)*
Lack of privacy in consultation spaces	*“… The consultation room itself is divided into three sections… this difficulty in guaranteeing privacy is what makes it difficult for us”* (KI.RR.002)
Caregiver disengagement and disclosure gaps negatively impacting retention and adherence to care	*“… many adolescents are on treatment*,* but they don’t know their status… Sometimes they are told to come and take the drugs … there are others that they say they don’t take them because that drug has no relevance… that is why often you can collect the viral load and find the viral load very high*,* it is because they don’t know they have and they don’t take it seriously and they take it looking like any medicine that is to be taken”* (HCW.MAR.002) *“…. the chaperones or parents of the adolescents I mean*,* that some have not disclosed their status to their children and so we have noticed some absences… If the father… doesn’t have time to accompany the son… he doesn’t show up because he doesn’t know what is going to be done there and his condition hasn’t been revealed yet”* (KI.ANG.003)
AYAHIV and caregivers’ poor HIV literacy	*“… the issue of knowledge*,* myths that exist in the community… can have a direct influence the health of the adolescent and young person…”* (KI.UU.002) *“… one of the main challenges is the lack of information for caregivers and most of the time we are talking about these adolescents and young people where at some point they need some caregiver to follow up on their treatment adherence*,* treatment care”* (KI.EE.001)
Limited access to health services	*“… we have very overcrowded AYFSs*,* at some point our AYFSs are not welcoming*,* since they lack places that would make the adolescent feel comfortable*,* and we have this issue of limiting the consultation time until 3 pm*,* we can’t go until 5 pm*,* and on Saturdays not all AYFSs are open and many AYAHIV study during the week”* (KI.GE.002) *“The other difficulty is the complains about transportation to get to the health facility*,* they live far away*,* so at some point…*,* one and another may be missing*,* because there is no money to come to the clinic*,* all these things create an impasse”* (HCW.AA.002)

Access constraints—such as limited clinic hours, transportation costs, and overcrowded facilities—were described as major barriers, particularly for adolescents balancing school schedules and treatment. Participants also described some inconsistencies between their service quality ratings and narrative accounts, which may reflect aspirational responses or social desirability bias. These findings point to both structural gaps and relational dynamics that limit service effectiveness across settings.

### Perceived efficacy of HIV services within AYFS to impact on retention, ART adherence, and stigma

Participants provided mixed perceptions on the effectiveness of the HIV services offered to AYAHIV within AYFS in improving retention, ART adherence and reducing stigma around HIV. While many acknowledged progress, particularly due to the integration of AYFS models, several challenges were noted.

#### Retention and ART adherence

Participants generally agreed that retention had improved with the creation of dedicated youth-friendly spaces, which allowed AYAHIV to engage without the discomfort of sharing adult queues.

*“In the olden days… the adolescents and young people would come to the health facility*,* and they would wait in the adult queue and at some point*,* they felt embarrassed because of their mentality. This constrained their retention in the services at some point. So*,* with the existence of AYFSs*,* retention improved because AYAHIV were among themselves and*,* no one felt fear about their presence there*,* differently from standing in the queue with adults”* (KI.II.002).

However, some participants shared the opinion that health services were still not able to effectively improve retention in care or adherence to ART. This was often hindered by stigma, nondisclosure of HIV status by caregivers, and logistical challenges such as transport and caregiver availability.

*“The services are not having much impact [in retention] …You have some adolescents who grow up and they ask why they are taking those pills and when the truth is not told they will feel the lack of such information. And if they don’t know why they are taking those pills*,* they don’t know the truth*,* and the health center and the health professional*,* they disclose it*,* but the mother I guess doesn’t want to feel guilty with the adolescent knowing why are taking pills and why they got the disease*,* so this leaves the teenager confused*” (KI.OO.002).

Notably, some participants appeared to conflate retention with ART adherence, often to appointment attendance as a proxy for adherence.

*“Services are having an impact on improving adherence to ART because of the strategy that we are using in these [HIV] appointments*,* they tend to see that they are reducing the number of dropouts and the number of the absentees that we are having”* (HCW.NN.002).

*“Yes*,* it is having an impact on the adherence because what you have to do is involve parents and caregivers because there were adolescents that many times you asked them why they were late to the HIV consultation and they responded*,* “Ah because I had no money for transportation”*,* or*,* “I had no way because my mother was away and it was very complicated”*,* but now this is improving”* (HCW.RR.002).

#### Stigma

Stigma and fear of disclosure were widely reported as ongoing barriers to adherence and engagement. Participants shared concerns that adolescents were reluctant to take medication or attend services for fear of being identified.

*“Well*,* [stigma and discrimination] is a challenge that we are struggling to overcome. We are succeeding but it is still a challenge because not everyone is accepting their [HIV] status*,* even to disclose to a friend”* (HCW.UU.001).

*“When diagnosed with HIV the teenager is afraid to disclose to others and end up not taking the medicine and not accepting because they are afraid to take that medicine and that their partner sees it…”* (KI.OO.002).

*“I even have one AYAHIV that until now is struggling*,* he still doesn’t want to adhere to the ART*,* but it is more out of fear. I talked to him*,* he said “I am afraid that my friends will find out*,* that is why I don’t take the pills”* (HCW.LL.002).

Despite this, few participants noted emerging signs of acceptance, especially among male AYAHIV, who were beginning to show increased comfort with their status as shown in the quote below:

“*Interviewer: What gives that impression that stigma is decreasing?*

*Interviewee: When I talk to some young men*,* I can see that they are already able to accept themselves and the disease that they have*,* they no longer feel that fear like they did after starting ART”* (HCW. II.002).

Also, some participants reported that HIV stigma was decreasing as some adolescents understood that they were not the only ones living with HIV, they were part of a large community of AYAHIV.

## Discussion

This study aimed to assess stakeholders’ perspectives and experiences concerning the provision of HIV services within AYFS in Nampula, Mozambique. While perceptions were generally positive, stakeholders reported substantial variation in service implementation across health facilities. The experiences of stakeholders highlighted eight key factors that served as facilitators to HIV service delivery for AYAHIV in health facilities within AYFS, including strong patient–provider relationships, collaborative care, well-trained HCWs and peers, support groups, caregiver support, effective dissemination of HIV awareness information, one-stop shops, and privacy within health facilities. At the same time, six major barriers were noted, such as inadequate staff training, staff shortages, limited privacy space, inconsistent caregiver involvement, insufficient HIV literacy among AYAHIV and caregivers, and access constraints. Notably, stakeholders expressed concern that existing services often fell short in meaningfully addressing persistent challenges related to ART adherence, retention in care, and stigma among AYAHIV.

### Variability in implementation of AYFS

Our study identified variations in perspectives among stakeholders regarding the pre-study implementation status of HIV care services for AYAHIV within AYFS in this setting. These differences may suggest disparities in services quality across health facilities, as the stakeholders’ assessments of HIV services were based on their experiences at the various affiliated facilities. However, with that said, the data also highlighted some inconsistencies between some participants’ numerical ratings of services and their narrative justifications of the scores. This misalignment may reflect social desirability or aspirational responses, particularly among stakeholders strongly committed to improving adolescent HIV care. On the other hand, some may have rated services optimistically based on perceived progress or future potential, despite acknowledging ongoing challenges. While these findings suggest potential differences in how AYFS are implemented across facilities, the inconsistencies between ratings and narrative responses limit the ability to draw definitive conclusions. This highlights the need for further systematic evaluation to assess the actual quality and scope of AYFS implementation and to determine whether perceived variability reflects real differences in service delivery. These recommendations are warranted given the well-recognized concern that services targeting youth are often poorly coordinated and uneven in quality [33]. Future research should consider building on these insights by formally assessing the quality of AYFS at facility level against the recommended standards. Such efforts could help identify areas within services that require improvement and ultimately provide an opportunity to standardize and further enhance HIV service delivery within AYFS in this setting.

### Facilitators and barriers to AYAHIV service delivery

This study revealed a set of interconnected factors influencing the delivery of HIV services within AYFS, reflecting both enabling conditions and persistent challenges. Key facilitators for service provision included patient-provider relationships, collaborative care, availability of well-trained HCWs and peers, support groups, caregiver engagement, effective dissemination of HIV awareness information; and structural innovations such as one-stop models and private consultation spaces. These facilitators did not operate in isolation, but rather worked in synergy to build trust, strengthen adolescent engagement, and improve continuity of care. Conversely, stakeholders identified a range barriers, including inadequate staff training, staff shortages, limited private spaces, inconsistent caregiver involvement, insufficient HIV literacy among AYAHIV and caregivers, and access constraints related to transport, clinic hours, and overcrowding. These barriers not only reflected structural gaps but also revealed deeper social and relational challenges—such as nondisclosure of HIV status and strained caregiver dynamics—that compromised adolescent autonomy and adherence. Notably, three factors—privacy, caregiver engagement, and HCW capacity— emerged as both barriers and facilitators to health service provision. This duality in categorization implies the unevenness in service provision across facilities and highlights critical leverage points for improving adolescent HIV care. The alignment of these findings with literature from other SSA contexts [13, 14, 18–20] reinforces the cross-cutting nature of these implementation challenges and opportunities. Moving forward, programs aiming to strengthen HIV services within AYFS should prioritize these domains to foster more consistent, adolescent-responsive service delivery.

### Implications for provider capacity and training

Our findings strongly support existing evidence for the need for comprehensive training among HCWs when it comes to caring for AYAHIV [20, 26]. Capacity building through training for HCWs has been closely linked to enhanced provider competence, confidence, and fidelity in providing AYFS [20, 34, 35]. The lack of training expressed by stakeholders is a matter of significant concern, and this gap was highlighted during the interviews. We observed some confusion among participants in distinguishing between retention and ART adherence in certain responses provided during the assessment of stakeholders’ perceptions about the impact of services on these HIV care outcomes. ART adherence refers to the extent to which a person takes antiretroviral medications as prescribed, while retention refers to the continued engagement of a person in HIV care over time. Several stakeholders used these terms interchangeably, likely without a clear perception and understanding of their distinctions. For instance, several respondents structured their responses concerning the influence of services on ART adherence by associating the retention of AYAHIV in care as a sign of their adherence to ART. Any confusion between these two may result in missed opportunities for identifying adherence challenges in patients who attend appointments but do not consistently take their medication. This is of particular concern, given the essential role HCWs play in facilitating the effective treatment of AYAHIV. This role encompasses monitoring their progress and identifying those requiring assistance when essential indicators deviate from expected levels. The importance of such efforts is heightened in instances where obstacles like non-acceptance of HIV status and resistance to ART are prevalent among AYAHIV. Furthermore, given reported staff shortages, it is plausible that gaps in understanding around HIV infection and treatment could be exacerbated by attempts to address this issue through the deployment of inadequately trained HCWs to fill these roles. Ironically, this approach might amplify barriers to the successful treatment and support of AYAHIV rather than mitigating them. Future work should evaluate the extent of the impact of staff shortages and turnover on the provision of HIV services for AYAHIV and explore suitable strategies to address the gaps introduced by these challenges.

### Addressing stigma, privacy, and service access

Our findings underscore that, although stakeholders believe that HIV services within AYFS positively impact retention, ART adherence, and stigma reduction among AYAHIV, these services fall short. This outcome was somewhat anticipated, given the challenges highlighted in the specific facilities. For instance, the absence of fundamental standards, such as private consultation spaces dedicated to AYAHIV, is likely hampering access to HIV services and perpetuating stigma. The importance of these factors in delivering quality care for AYAHIV is widely recognized [14, 20, 35]. While there is some optimism evident in reports of incremental progress, it remains imperative to address these barriers comprehensively for these services to exert a significant impact on these outcomes. This assessment highlights the relevance of research studies, such as CombinADO, in this setting, focusing on developing and testing multicomponent adolescent and youth-specific intervention strategies to comprehensively address challenges and enhance service delivery. Such efforts could encompass ongoing training programs for HCWs, offering supportive supervision and monitoring, infrastructure improvements, and community engagement initiatives to reduce stigma. These components were essential for implementing the CombinADO intervention.

Our study has certain limitations that deserve acknowledgement. The first limitation lies in the qualitative nature of our research, raising the possibility that the researcher’s subjectivity may have influenced the analysis and interpretation of the data. Nevertheless, we mitigated this potential bias by involving two well-trained qualitative analysts who guided all stages of the analysis and interpretation of results. Second, we recognize that the captured viewpoints might not be transferable to other contexts, due to the qualitative design used and further compounded by non-responses to certain questions from a few stakeholders, likely from a lack of understanding of these questions. Nonetheless, we emphasize that the insights presented here are derived from stakeholders working across a diverse range of health facilities. This includes facilities of varying sizes and locations, encompassing both urban and rural settings, which adds substantial value to the findings. In addition, we provide enough details about the study setting and context to allow for readers to assess the applicability of the findings to their specific situation. Third, it is important to note that this sub-study did not include perspectives from AYAHIV attending clinics where data were collected. However, their input was incorporated into the broader CombinADO study through a participatory human-centered design approach during the development of the CombinADO intervention. Their perspectives will also be explored further in future research components. Despite the above limitations, the present exploratory phase offers valuable insights into service delivery from the perspectives of implementers and contributes to identifying areas of improvement in HIV service provision within AYFS. While not exhaustive, this study sets the groundwork for future research in this area. Our findings extend the implementation science literature on youth-friendly HIV care in sub-Saharan Africa by offering stakeholder-based insight into multilevel barriers and enablers in the Mozambican context. These insights are critical for refining intervention strategies like CombinADO and enhancing scalability and sustainability in similar settings.

## Conclusions

Stakeholders shared positive perceptions and experiences regarding delivering HIV services to AYAHIV within the existing AYFS in Nampula. However, they also identified barriers that hinder the effectiveness of these services in addressing challenges related to retention, ART adherence, and HIV stigma among AYAHIVs. Particularly, our findings emphasize the importance of comprehensive and continuous capacity-building for HCWs, suggesting a potential need to reassess the current training model and incorporate ongoing training activities to enhance understanding of key HIV concepts. Moreover, addressing concerns related to staff turnover as well as ensuring the availability of private spaces for delivering adequate HIV services for AYAHIV may be necessary within health facilities offering AYFS in this context. These endeavors may enhance the quality and effectiveness of HIV services delivered to AYAHIV that are provided within AYFS, ultimately contributing to improved HIV outcomes in this population. Notably, findings from this study were used to inform the refinement of the CombinADO intervention package, which incorporated enhanced HCW training, infrastructure strengthening, and peer-led community engagement to more effectively respond to the needs of AYAHIV accessing HIV services within AYAFS in Nampula.

## Supplementary Information

Supplementary Material 1.

## Data Availability

Data collection tools have been provided within the supplementary information files, any additional material can be requested from the corresponding author or contacts listed in ClinicalTrials.govNCT04930367.

## Abbreviations

ART: Antiretroviral Therapy
AYAHIV: Adolescents and Young Adults Living with HIV
AYFS: Adolescent and Youth Friendly Services
HCW: Health Care Worker
KI: Key Informant
LMIC: Low and Middle-Income Countries
SSA: Sub-Saharan Africa
