# Supplementary material for: A qualitative evaluation of stakeholder perspectives on the implementation of HIV services within adolescent and youth-friendly services for the youth in Nampula, Mozambique
Source: BMC Health Serv Res. 2025 Sep 30;25:1229. doi: 10.1186/s12913-025-13414-0 (PMC12482722; doi:10.1186/s12913-025-13414-0)
Supplement: Supplementary file 1 — Supplementary Material 1. [file 12913_2025_13414_MOESM1_ESM.pdf]

Supplementary 1: Qualitative interview guides for stakeholders included in the pre-implementation phase of the CombinADO study

Healthcare Providers Interview Guide

FOR INTERVIEWER: **Healthcare Providers might include nurses, medical doctors and counsellors who are directly involved in the implementation of HIV services for AYAHIV.**

INTERVIEWER READS**:** Now I would like to get some additional information on your experience implementing and supervising the implementation of HIV services for AYAHIV. In this interview, there are no right or wrong answers; your opinions and experiences in your own words are what is important. You do not have to answer questions you do not feel comfortable discussing.
As a reminder, the information that you share during this interview will be kept private. With your permission, this interview will be audio-recorded. The audio recording and transcripts will be labelled with a participant ID number only.

1) Overall, how is the implementation of HIV services for AYAHIV going in this HF?
a) On a scale of 1 to 10, where 1 is not well at all, and 10 is very well.
b) What is working well? What components work well?
c) What is not working well? Why?
d) How could the services be improved? What changes would you recommend?

2) Do you think that the HIV services for AYAHIV will have/had an impact on the following:
a) Improve retention in care for AYAHIV? Why or why not?
b) Improve adherence to ART for AYAHIV? Why or why not?
c) Help to address stigma around HIV? Why or why not?

3) Are there specific groups of AYAHIV- pregnant girls, non-pregnant girls/boys, older/younger adolescents that you think are benefiting specifically from the services? Why do you think that these groups benefit?

4) What are the main factors that facilitate the implementation of HIV services for AYAHIV?

5) What are the main challenges to the implementation of HIV services for AYAHIV?

6) Is there anything else you would recommend improving the HIV services for this population?

Thank you very much for participating in this interview and sharing your thoughts and experiences with me.

Key Informants Interview Guide

***FOR INTERVIEWER:* Key Informants might include ICAP staff and health facility managers involved in the implementation, management and supervision of HIV services for AYAHIV.**

***INTERVIEWER READS*:** Now I would like to get some additional information on your experience implementing and supervising the implementation of HIV services for AYAHIV. In this interview, there are no right or wrong answers; your opinions and experiences in your own words are what is important. You do not have to answer questions you do not feel comfortable discussing.
As a reminder, the information that you share during this interview will be kept private. With your permission, this interview will be audio-recorded. The audio recording and transcripts will be labelled with a participant ID number only.

1) Overall, how is the implementation of HIV services for AYAHIV going in this HF?
a) On a scale of 1 to 10, where 1 is not well at all, and 10 is very well.
b) What is working well? What components work well?
c) What is not working well? Why?
d) How could the services be improved? What changes would you recommend?

2) Do you think that the HIV services for AYAHIV will have/had an impact on the following:
a) Improve retention in care for AYAHIV? Why or why not?
b) Improve adherence to ART for AYAHIV? Why or why not?
c) Help to address stigma around HIV? Why or why not?

3) Are there specific groups of AYAHIV- pregnant girls, non-pregnant girls/boys, older/younger adolescents that you think are benefiting specifically from the services? Why do you think that these groups benefit?

4) What are the main factors that facilitate the implementation of HIV services for AYAHIV?

5) What are the main challenges to the implementation of HIV services for AYAHIV?

6) Is there anything else you would recommend improving the HIV services for this population?

Thank you very much for participating in this interview and sharing your thoughts and experiences with me.
